# Monocyte Chemotactic Protein-Induced Protein 1 (MCPIP-1): A Key Player of Host Defense and Immune Regulation

**DOI:** 10.3389/fimmu.2021.727861

**Published:** 2021-10-01

**Authors:** Zhuqing Jin, En Zheng, Candice Sareli, Pappachan E. Kolattukudy, Jianli Niu

**Affiliations:** ^1^ School of Basic Medical Sciences, Zhejiang Chinese Medical University, Hangzhou, China; ^2^ Department of Chemistry, Zhejiang University, Hangzhou, China; ^3^ Office of Human Research, Memorial Healthcare System, Hollywood, FL, United States; ^4^ Burnett School of Biomedical Sciences, University of Central Florida College of Medicine, Orlando, FL, United States

**Keywords:** inflammation, viral infection, ischemic inflammation, resolution, MCPIP-1

## Abstract

Inflammatory response is a host-protective mechanism against tissue injury or infections, but also has the potential to cause extensive immunopathology and tissue damage, as seen in many diseases, such as cardiovascular diseases, neurodegenerative diseases, metabolic syndrome and many other infectious diseases with public health concerns, such as Coronavirus Disease 2019 (COVID-19), if failure to resolve in a timely manner. Recent studies have uncovered a superfamily of endogenous chemical molecules that tend to resolve inflammatory responses and re-establish homeostasis without causing excessive damage to healthy cells and tissues. Among these, the monocyte chemoattractant protein-induced protein (MCPIP) family consisting of four members (MCPIP-1, -2, -3, and -4) has emerged as a group of evolutionarily conserved molecules participating in the resolution of inflammation. The focus of this review highlights the biological functions of MCPIP-1 (also known as Regnase-1), the best-studied member of this family, in the resolution of inflammatory response. As outlined in this review, MCPIP-1 acts on specific signaling pathways, in particular NFκB, to blunt production of inflammatory mediators, while also acts as an endonuclease controlling the stability of mRNA and microRNA (miRNA), leading to the resolution of inflammation, clearance of virus and dead cells, and promotion of tissue regeneration *via* its pleiotropic effects. Evidence from transgenic and knock-out mouse models revealed an involvement of MCPIP-1 expression in immune functions and in the physiology of the cardiovascular system, indicating that MCPIP-1 is a key endogenous molecule that governs normal resolution of acute inflammation and infection. In this review, we also discuss the current evidence underlying the roles of other members of the MCPIP family in the regulation of inflammatory processes. Further understanding of the proteins from this family will provide new insights into the identification of novel targets for both host effectors and microbial factors and will lead to new therapeutic treatments for infections and other inflammatory diseases.

## Introduction

Inflammatory response is an immunological defense mechanism of the host to infections and tissue damage (e.g., ischemic insults) or stress (1). Damaged or stressed cells are thought to release danger-associated molecular patterns (DAMPs) that can trigger the innate immune system in the same manner as microbial components, the so-called pathogen-associated molecular patterns (PAMPs), by binding to host pattern-recognition receptors (PRRs) presented on various cells, including immune cells (T-cells, B-cells and NK cells) and the tissue cells such as endothelial cells, cardiomyocytes, and even neurons (1). This recognition triggers a series of signaling cascades that initiate the activation of transcriptional factor nuclear factor-κB (NFκB) and NLRP3 inflammasome, leading to the release of inflammatory mediators, like tumor necrosis factor-alpha (TNFα), monocyte chemoattractant protein-1 (MCP-1), interleukin-1 (IL-1) and IL-6 (2, 3). The inflammatory response is beneficial to the host by eliminating the harmful agents (PAMPs or DAMPs) and is usually resolved in a timely manner; however, failure to resolve can cause excessive or persistent inflammation that is often disruptive and can cause marked tissue damage (4).

The resolution of inflammation is a programmed active process that involves the biosynthesis of a variety of active molecules that act on key events of inflammatory response to terminate the production of pro-inflammatory mediators and restore tissue homeostasis (5). There is an increasing body of evidence that many pro-inflammatory mediators produced during the inflammatory phase can simultaneously initiate a program for active resolution (6). For example, TNFα can effectively induce A20 (also known as tumor necrosis factor-α-induced protein 3 or TNFAIP3), an ubiquitin editing enzyme that negatively regulates the inflammatory response by interfering with NFκB signaling pathway, leading to the resolution of inflammation (7). The newly synthesized molecules not only act as signals for the termination of the inflammatory response, but also promote the clearance of dead cells to accelerate the resolution of inflammation (4, 8).

A major contribution of our group to the field was the discovery of the novel zinc-finger protein, named MCP-1–induced protein (MCPIP), which was originally detected in MCP-1 treated human peripheral blood monocytes (9, 10). Subsequent studies demonstrated that MCPIP belongs to a new Zc3h12 family consisting of four members (MCPIP-1, -2, -3, and -4) that are encoded by Zc3h12a, Zc3h12b, Zc3h12c, and Zc3h12d, respectively (11). MCPIP-1 is the most-studied protein that contains an N-terminal domain, a PilT N-terminus like (PIN) domain, a zinc finger domain, and a C-terminal domain (12). Emerging evidence indicates that MCPIP-1 plays an essential role in the regulation of inflammatory response, with additional roles in defense against viruses and various stresses, cellular differentiation, and apoptosis (13, 14), all of these are key cellular and molecular components that contribute to the successful resolution of inflammation (9, 10, 15–18). The focus of this review is to present evidence illustrating that the role played by MCPIP-1 is important in the resolution of inflammation initiated by virus infections or ischemic injuries and highlight recent advances on the actions of this protein and its potential clinical significance. We also discuss the available evidence regarding the role of other members (MCPIP-2, -3, and -4) from this family in the regulation of inflammatory processes.

## Expression and Dynamic Regulation of MCPIP-1

MCPIP-1 was originally identified in human peripheral blood monocytes stimulated with MCP-1 (9). Subsequent studies demonstrated that MCPIP-1 is produced in many other cell types, either constitutively or after induction by a wide range of stimuli, such as inflammatory cytokines (e.g., IL-1β, IL-17 and TNFα) and oxidative stress (13, 19, 20). Further studies demonstreated that the expression of MCPIP-1 can be induced by ischemia in the heart and the brain (21, 22). Infections by virus such as hepatitis C virus (HCV), hepatitis B virus (HBV), influenza A virus (IAV), Japanese encephalitis virus (JEV) and Dengue virus as well as by bacteria and fungal increase the expression of MCPIP-1 (23–25). The extracellular high mobility group box 1 (HMGB1), a non-histone DNA-binding protein released from dying cells in response to tissue injuries, also increases microglium expression of MCPIP-1 that negatively regulates HMGB-1-mediated neuroinflammation and neuronal toxicity (26). Minocycline, a member of tetracycline antibiotics with anti-inflammatory properties, also induces MCPIP-1 expression in the heart and the brain (27, 28).

Although the molecular mechanisms responsible for MCPIP-1 expression are still poorly understood, MCP-1 binding to its cognate receptor CCR2 was thought to activate ERK or AKT pathways, leading to the expression of MCPIP-1 (9). Activation of NFκB signaling was suggested to induce the expresion of MCPIP-1 by inflammatory cytokines such as IL-1β (29). Transcription factors Elk-1 and SRF were also reported to mediate IL-1-dependent expression of MCPIP-1 by binding to the promoter region of MCPIP-1 (30). Activation of JAK/STAT3 signaling was also reported to mediate MCPIP-1 expression in epithelial cells (31). At the post-transcriptional level, MCPIP-1 mRNA was found to be downregulated by miR-9 in LPS-activated microglial cells (32). MCPIP-1 also cleaves its own transcript (33, 34). Moreover, the translated MCPIP-1 protein can be phosphorylated by IκB kinase (IKK) β and then undergoes ubiquitination and degradation (35) or cleaved by the paracaspase Malt-1 (33, 36). These data indicate that MCPIP-1expression is tightly controlled by an autoregulatory feedback mechanism, which ensures an appropriate level of MCPIP-1 aimed to minimize any disruption of immune homeostasis.

## Functional Features of MCPIP-1

MCPIP-1 was first described as a transcriptional activator owing to the structural feature of a potential DNA binding zinc finger domain (9). Subsequent studies indicate that MCPIP is localized to both the cytoplasmic and nuclear compartments, depending on the distinct functions it plays in different cell types (11, 37, 38). Mutational analysis of MCPIP-1 has identified the two regions of the primary structure that are critical for its biological activity (12, 39–41). The first region consists of the ubiquitin-associated domain from 43-89, which is associated with the control of protein ubiquitination; whereas the second region consists of PIN domain from 133–270, which is associated with RNA-cleaving function and is why it was later renamed as Regnase-1 (regulatory RNase-1) (40, 42). In addition to these two regions, the C-terminal region of the NYN domain is crucial for suppressing microRNA (miRNA) biogenesis *via* cleavage of the terminal loops of precursor miRNAs (16, 40–44).

### MCPIP-1 Regulates Protein Ubiquitination

**Figure 1:**
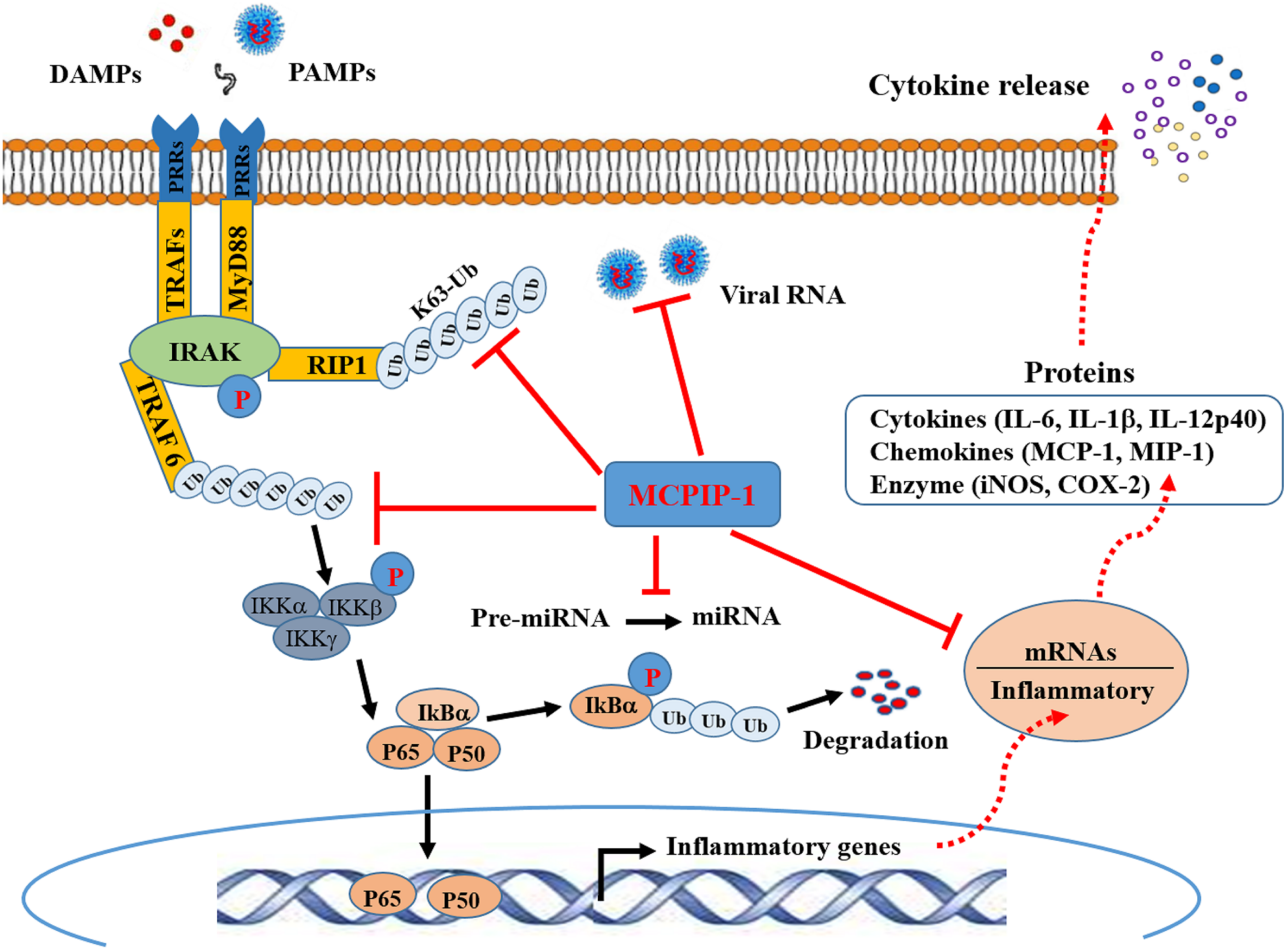
Schematic representation of anti-inflammatory activity of MCPIP-1. Binding of molecules (DAMPs) derived from tissue damage or pathogens (PAMPs) to PRRs triggers interactions between the cytoplasmic adaptor proteins and the kinases IRAK. This engages the ubiquitin ligase TRAF6 to make polyubiquitin chains that activate the IKK complex, leading to phosphorylation and subsequent ubiquitination IKBα. This releases P50/p65 dimer for entry into the nucleus to cause transcriptional activation of NFκB-dependent genes encoding inflammatory cytokines. Ubiquitylation of RIP1, and potentially other components of the complex, recruits IKKγ and TAK1 for NFκB and MAPK activation (not shown). MCPIP-1 hydrolyzes all of these K48- and K63-linked polyubiquitins to block NFκB activation. The RNase activity of MCPIP-1 also degrades viral RNA and some mRNAs encoding for inflammatory cytokines, leading to dampening of protein expression of the inflammatory cytokines. The anti-Dicer activity of MCPIP-1 can cleave the terminal loops of pre-miRNAs leading to destabilization of pre-miRNAs and suppression of the miRNA biogenesis.

Ubiquitination has emerged as a crucial mechanism that regulates signal transduction in the inflammatory response (3, 45). The use of MCPIP-1-deficient mice has revealed the crucial role of MCPIP-1 in the regulation of inflammatory cytokine signaling pathways. Mice lacking MCPIP-1 are normal at birth but suffer growth retardation and die prematurely due to massive multi-organ inflammation, indicative of a key role for MCPIP in immune homeostasis of the host (41). Macrophages from MCPIP-1-deficient mice showed up-regulation of pro-inflammatory mediators together with a greatly increased ubiquitination of TRAFs (TNF receptor–associated factors) and the receptor-interacting protein (RIP) kinases, both of which play a central role in the LPS-, IL-1β- and TNF-induced activation of NFκB signaling pathway (46). The purified MCPIP-1 protein was shown to cleave K48- or K63-linked polyubiquitin chain, while this action of MCPIP-1 was inhibited by N-ethyl maleimide, a known inhibitor of cysteine proteinases (41). Consistent with this finding, treatment of high-molecular-weight K63-linked polyubiquitin with purified MCPIP-1 caused hydrolysis of the polyubiquitin, leading to the inhibition of phosphorylation of TAK1 (47), a critical factor for activation of the downstream kinase IKK, thereby mediating IκBα phosphorylation and NFκB activation (48). Accordingly, deletion of the ubiquitin association domain of MCPIP-1 resulted in the loss of inhibition of TNFα-induced NFκB activation (41). These results indicate that MCPIP-1 can act as a deubiquitinase to hydrolyze K63-, K48-linked polyubiquitin chains and inhibits NFκB transcriptional activity, thus contributing to suppression of the pro-inflammatory response (**Figure 1**). MCPIP-1 has also been reported to stabilize NFκB essential modulator by promoting deubiquitination, resulting in subsequent inhibition of NFκB activation induced by DNA damage (49). MCPIP-1 was also shown to stabilize the hypoxia-inducible factor 1alpha protein that is required for macrophage maturation under hypoxic conditions, in which deubiquitination plays a key regulatory role (50).

### MCPIP-1 Regulates Inflammatory mRNA Stability

MCPIP-1 can also act as an RNase to regulate mRNA stability (19, 33, 40, 51). MCPIP-1 can directly bind the 3′-untranslated region (UTR) of IL-6 mRNA and manifested RNase activity to degrade IL-6 transcripts (40). A similar finding was demonstrated in the expression of IL-1β mRNA that was degraded by the increased level of MCPIP-1 in HepG2 and U937 cells (19). Genome-wide association studies showed that the PIN domain of MCPIP-1 contains the RNase catalytic center that requires an interaction with the N-terminus for its full RNase activity and the zinc-finger domain is responsible for the recognition and direct binding of the mRNAs (12, 39, 51). With the cooperation of its domains, MCPIP-1 recognizes and degrades target mRNAs by recognizing stem-loop structures at the 3′-UTRs of these genes (40, 51). In T cells, MCPIP-1 downregulates a set of genes by cooperating with roquin, another CCCH type zinc finger protein (36, 51). However, a recent study in other type of cells indicates that, although roquin and MCPIP-1 control shared mRNAs, they do so in different mechanisms within different subcellular compartments (51). Thus, the co-operation between MCPIP-1 and roquin remains incompletely understood. In different cell types, MCPIP-1 recognizes and degrades target mRNAs may be cell-specific.

To date, MCPIP-1-dependent degradation of inflammatory mRNAs has been increasingly identified. In addition to IL-6 and IL-1β, mRNAs encoding for IL-2, IL-12 and IL-17 have been identified as direct targets of MCPIP-1 (51, 52). Both CXCL1 and CXCL2, two important chemokines contributing to early stage neutrophil recruitment during tissue inflammation (53), are also direct targets of MCPIP-1 (51). MCPIP-1 also degrades mRNAs encoding T-cell co-stimulatory receptors such as ICOS, TNFR2 and OX40 as well as T-cell activation marker CD44 (33), all of which play a key role in permitting T cell mature and activation. Importantly, MCPIP-1 degrades mRNA encoding for the anti-apoptotic immediate early response 3 (IER3) protein (54), resulting in apoptosis of macrophages that contributes to resolution of inflammation. Thus, MCPIP-1 controls inflammatory response not only by preventing the transcription of the inflammatory cytokines, but also by dampening of the protein expression of the inflammatory cytokines at the post-transcriptional level as well (**Figure 1**).

### MCPIP-1 Regulates miRNA Processing

Emerging studies have shown that miRNAs modulate many aspects of the immune responses such as proliferation, differentiation, cell fate determination, immune cell function, and cytokine responses (55, 56). when miRNAs are aberrantly expressed they contributes to the pathogenesis of inflammatory and autoimmune diseases by regulating their cellular and molecular targets (55). Besides targeting mRNA, MCPIP-1 was shown to regulate miRNA biogenesis by counteracting Dicer, a central ribonuclease in miRNA biosynthesis (42, 57). MCPIP-1 can cleave the terminal loop of pre-miRNAs, thereby inhibiting their maturation (41). Studies have shown that miRNA-146a and miRNA-155 are specifically down-regulated by MCPIP-1 (42, 58). Both miR-146a and miR-155 have been proposed to regulate the macrophage activation by forming a combined negative and positive regulatory loop that alters NFκB activity (59). miR-155 is highly transcribed upon an inflammatory stimulus, which can amplify NFκB activity, while as an inflammatory response develops, miR-146a levels accumulate, which causes suppression of IRAK1 and TRAF6, leading to the inhibition of NFκB activation (59). However, a study by Mino et al. indicated that expression of both miR-155 and miR-146 was not altered in mouse embryonic fibroblasts from MCPIP-1-deficient mice (51). Therefore, MCPIP appears to utilize distinct mechanisms to keep the inflammatory signaling suppressed and to re-establish immune hemostasis (**Figure 1**).

### The Pro-Apoptotic Activity of MCPIP-1

Apoptosis is an evolutionarily conserved cell death program that is tightly regulated by the Bcl-2 family of proteins, which contains both pro-apoptotic and pro-survival members that balance the decision between cellular life and death (60). Microarray analysis revealed that MCPIP-1 upregulates the pro-apoptotic genes and downregulates the anti-apoptotic genes in the myocardium (9). Along this line, MCPIP-1 was found to mediate endothelial cell apoptosis and dysfunction upon MCP-1 treatment (61). The pro-apoptotic activity of MCPIP-1 was further documented *in vitro* assays in HEK 293 cells (9), H9c2 cardiomyoblasts (62), neonatal rat cardiomyocytes (63), macrophages (64, 65), neutrophils (66), T cells (67), and even cancer cells (68). Mechanistically, MCPIP-1 selectively binds and cleaves the mRNAs of anti-apoptotic genes, such as Bcl-2A1, Bcl-2L1, and RELB, leading to down-regulation of anti-apoptotic proteins and upregulation of pro-apoptotic proteins (66, 68). The inhibition of miRNA biogenesis by MCPIP-1 is also linked to its pro-apoptotic activity. MCPIP-1 has been shown to downregulate miR-3613-3p expression in neuroblastoma cells, which in turn upregulates apoptotic protease activating factor 1, causing apoptosis by caspase-9 proteolysis (69). The pro-apoptotic activity of MCPIP-1 was found to be associated with its influence on the formation of stress granules (SGs), one kind of non-membranous ribonucleoprotein complexes containing untranslated mRNA formed in response to stress exposure (70). MCPIP-1 can completely block SG formation and promote macrophage apoptosis (71).

It is well documented that the amount of reactive oxygen species (ROS) produced and the extent of oxidative stress in a cell determine the fate of the cell to die or survive (72). The pro-apoptotic activity of MCPIP-1 was strongly correlated with its ability to induce intracellular ROS that cause endoplasmic reticulum (ER) stress, resulting in autophagy and apoptosis in cardiomyocytes (62, 63), macrophages (64, 73), endothelial cells (74, 75), and in renal cell carcinoma (76). Deubiquitination of RIP1 by CYLD or A20 has been suggested to facilitate cell death (77, 78). As a new member of the deubiquitinase family, however, the role of the deubiquitinating activity of MCPIP-1 in cell death remains to be determined. While it has been reported that MG-132, a proteasome inhibitor, effectively upregulates MCPIP-1 expression, potently activating the apoptosis of cancer cells (79). Further research is needed to fully understand its significance in regulating cell death.

## Roles of MCPIP-1 in the Resolution of Inflammation

**Figure 2:**
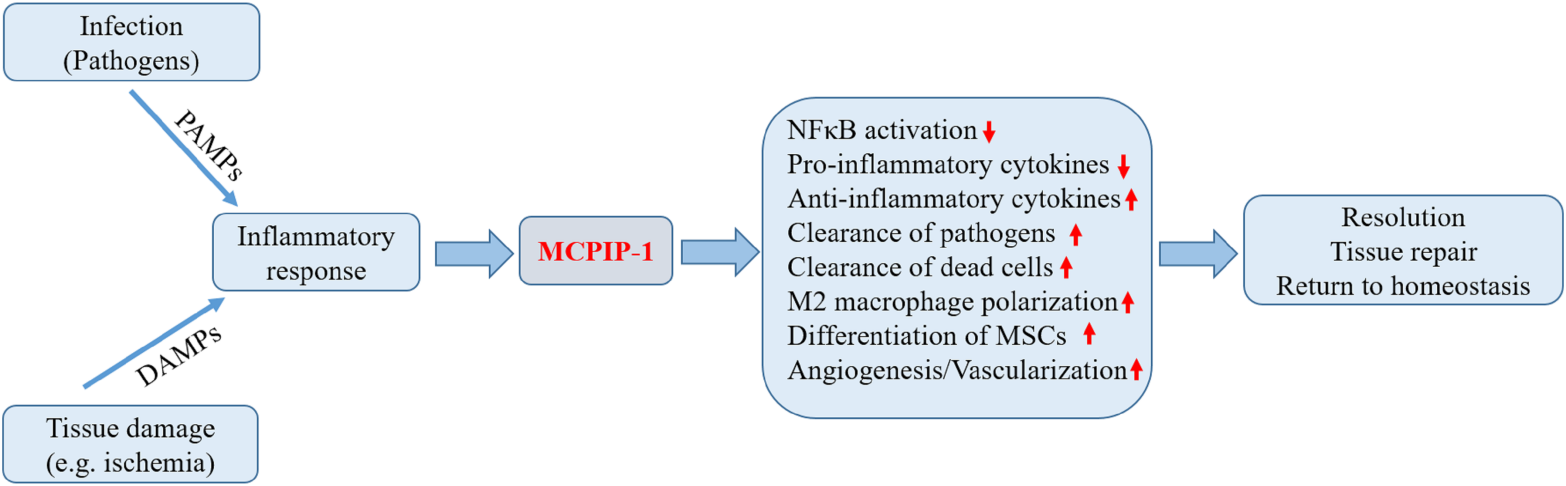
Biological roles of MCPIP-1 associated with resolution of inflammation. During the early phase of inflammation, inflammatory response initiated by infection or tissue injury activates the endogenous defense mechanisms aimed to bring about proper resolution. The expression of MCPIP-1 induced by the inflammatory response modulates a wide range of cellular and molecular events associated with the resolution of inflammation. The expression of MCPIP-1 results in the suppression of NFκB activation and reduced pro-inflammatory cytokines. MCPIP-1 is able to induce apoptosis of infected or damaged cells, leading to their clearance by macrophages. In addition, MCPIP-1 promotes M2 macrophage polarization and enhances angiogenic differentiation of mesenchymal stem cells (MSCs). These events will create a favorable environment contributing to the resolution and tissue homeostasis.

Resolution of inflammation is a coordinated and active process that involves the suppression of pro-inflammatory reaction, apoptosis and subsequent clearance of activated inflammatory cells, and repolarization of macrophages towards a resolving phenotype aimed at restoration of tissue integrity and function (5, 6). As mentioned above, the functions of MCPIP-1 support the notion that MCPIP-1 has critical roles in restricting inflammation (**Figure 2**). In the following we present the experimental findings to provide an overview of MCPIP-1 that drives these resolution processes.

### MCPIP-1 Restricts Virus Replication and Inflammation

Acute inflammation occurs in response to pathogen infection. This process involves the activation of innate responses that enhance innate microbial killing and clearance to protect organ structure and function (80). MCPIP-1 has been evident in cells infected with various viruses and exerts antiviral activity (23–25, 81, 82). MCPIP-1 can distinguish mRNAs from the host genome and can selectively degrade foreign mRNAs through its RNase activity (83). Indeed, MCPIP-1 is activated upon viral infections and has been shown to restrict virus replication by directly binding and subsequently degrading viral RNAs, such as JEV, IAV, DEN, coxsackievirus B3, encephalomyocarditis virus, and HIV (23, 81, 84). The deubiquitinase activity has not been shown to play a role in the antiviral effect of MCPIP-1, even though ubiquitination has been implicated in virus replication (85, 86). MCPIP-1 also restricts HCV replication by directly degrading HCV RNA and inhibits HCV-mediated expression of pro-inflammatory response (23, 24). In patients with chronic hepatitis C, the expression of MCPIP-1 in the liver has been suggested to have a protective role in antiviral responses (87). Type I interferons (IFNs) are recognized as the first line of defense against viral infection (88). MCPIP-1 has been proven to be a positive feedback amplifier of IFN signaling and promotes innate antiviral immunity independently of its RNase and deubiquitinase activities (82). Therefore, MCPIP-1 seems to be a very promising target for antiviral infections by degrading viral genomes, suppressing virus-mediated expression of pro-inflammatory cytokines or through the induction of antiviral effector molecules, resulting in the resolution of inflammation. These findings also reveal an exciting possibility for MCPIP-1 protecting against the infection caused by the SARS-CoV-2, an RNA virus that may cause severe acute respiratory distress syndrome due to “cytokine storms” induced by a hyper-activation of inflammatory cytokine response (89, 90).

### MCPIP-1 Regulates Post-Ischemic Inflammation

Inflammatory response represents one of the first immune processes following ischemic injury, which is usually self-limiting, followed by tissue repair and healing responses (91, 92). Ischemic preconditioning is a well-established phenomenon, in which brief episodes of sub-lethal ischemia and reperfusion elicit strong cellular protection against the subsequently sustained ischemia in the heart and the brain (93, 94). There is increasing evidence that ischemic preconditioning induces a powerful anti-inflammatory response (94–96), which has been well illustrated by ‘endotoxin tolerance’ and is thought to be an adaptive response conferring protection by suppression of the hyper-activation of the innate immune system through auto-regulatory network of cytokines (94, 97). Preconditioning stimuli with low doses of LPS, a primary ligand for TLR4, provides protection against subsequent challenges with injurious focal ischemia in the brain that is similar to ischemic preconditioning (98). Diminished activation of cellular inflammatory responses to ischemia by LPS preconditioning has been suggested to play an important role for protection against ischemic injury (98, 99). In a mouse model of middle cerebral artery (MCA) occlusion, LPS induces upregulation of MCPIP-1 in the brain and the neuroprotection offered by LPS preconditioning was diminished due to MCPIP-1 deficiency, suggesting MCPIP-1 expression initiated by LPS preconditioning may be an intrinsic cellular defense mechanism against the sustained ischemic injury (100). Consistently, mice lacking MCPIP-1 showed an enhanced leakage of blood–brain barrier, increased production of cytokines and a larger infarct volume after MCA occlusion (21, 101). This scenario was further demonstrated in the heart, where cardiac-specific expression of MCPIP-1 protected against LPS-induced myocardial inflammation and dysfunction (44). Consistently, mice with cardiac-specific expression of MCPIP-1 showed cardioprotective effects against myocardial infarction, as evidenced by the improved cardiac function, mitigated interstitial myocardial fibrosis, increased apoptosis of inflammatory cells, and decreased myocardial inflammation (22). In cardiomyocytes, NFκB activity was increased in response to LPS but suppressed by forced expression of MCPIP-1, thus linking MCPIP-1 to the suppression of myocardial inflammation in response to cardiac stress (22, 44). Consistent with this findings, NFκB activity initiated by myocardial infarction was inhibited by forced expression of MCPIP-1, suggesting that the preconditioning-like effects of MCPIP-1 probably involve its ability to inhibit NFκB activation (22, 44). The murine hearts expressing MCPIP-1 also displayed lower expression of inflammation-associated miR-126,-146,-155 and -199a when compared to those seen in the wild type mice (22). Preconditioning by minocycline is a pharmacological alternative to ischemic preconditioning, which enhances neuroprotection after ischemic stroke (102). Minocycline preconditioning inhibits the inflammatory cytokine response to ischemia through preferential induction of MCPIP-1, and the protective role of minocycline was diminished in the MCPIP-1-deficient mice subjected to focal cerebral ischemia/reperfusion (I/R) injury (27). Similar findings were observed in a mouse model of myocardial I/R injury, in which minocycline attenuated myocardial I/R injury *via* upregulating MCPIP-1 that subsequently inhibited NFκB activation and pro-inflammatory cytokine secretion (28). These findings are in agreement with the previous reports that MCPIP-1 deficiency results in massive multi-organ inflammation and premature death in mice (40, 41, 43).

The mechanisms underlying the preconditioning-like cellular protection by MCPIP-1 need further investigation. MCPIP-1 is likely to alter the immune responses to the ischemic insults by limiting pro-inflammatory cytokine transcription, dampening of protein expression of pro-inflammatory cytokines, regulating synthesis of pro-inflammatory miRNAs or enhancing clearance of the infiltrated inflammatory cells (44, 100). The combination of these mechanisms may result in an effective resolution of inflammation, pointing to MCPIP-1 as a promising target for the development of new therapeutic strategies to treat post-ischemic inflammation, and therefore warrant future studies on the molecular mode of action of MCPIP-1 in inflammatory diseases.

### Pleiotropic Effects of MCPIP-1 On Inflammation

Resolution of inflammation is a coordinated process that requires a tight interplay between macrophages, stem and progenitor cells, together with stromal cells to restoration of tissue integrity and function (5, 6). Beyond its anti-inflammatory activity, MCPIP-1 has also shown some beneficial pleiotropic effects, contributing to the resolution of inflammation and the restoration of tissue homeostasis.

MCPIP-1 was shown to induce angiogenesis by promoting the migration and apoptosis of human umbilical vein endothelial cells (HUVECs) and the expression of angiogenesis-related gene CDH12 and CDH19 (103). Moreover, MCPIP-1 inhibits the production of anti-angiogenetic miR-20b and miR-34a, which repress the translation of HIF-1α and SIRT-1 respectively, leading to promoting angiogenesis in the HUVECs (50). These findings agree with the animal data showing that forced expression of MCPIP-1 induces angiogenesis of bone marrow monocytic cells and accelerates post-ischemic neovascularization (104). Mesenchymal stem cells (MSCs) are candidates for cellular therapies aimed at promoting tissue repair or immunoregulation (105). MCPIP-1 was shown to increase angiogenic and cardiac differentiation capacity of bone marrow-derived MSCs, contributing to repair and regeneration of ischemic myocardium (106). Vascular endothelial and smooth muscle cells play critical roles in the stability and tonic regulation of vascular homeostasis. MCPIP-1 was shown to regulate the phenotypic switching of both endothelial and smooth muscle cells *via* suppression of synthesis of miRNAs, such as miR-126, 145, -146a, and -223 (43, 107). However, Marona et al. reported that MCPIP-1 reduces tumor vascularity in clear cell renal cell carcinoma by inhibiting the recruitment of bone marrow-derived endothelial progenitor cells (EPCs) and phosphorylation of VE-cadherin *via* the degradation of mRNAs encoding for IL8, VEGF and CXCL12 (108).

A key event required for resolution of inflammation is efferocytosis of apoptotic and necrotic cells, mostly by macrophages acquiring an alternative M2 phenotype (109). We reported the ability of MCPIP-1 to control macrophage reprogramming toward a M2 phenotype, resulting in reduced production of pro-inflammatory cytokines and increased release of anti-inflammatory and reparative mediators (16). With MCPIP-1 mutants that have only one of the two catalytic activities, both the deubiquitinase and RNase activities of MCPIP-1 were shown to play a critical role in M2 macrophage polarization (16, 110). MCPIP-1was also reported to suppress the synthesis of miR155 and upregulate miR-223 and miR-146 expression, contributing to M2 polarization (111). By its RNase activity, MCPIP-1 is capable of suppressing the expression of a group of mRNAs encoding factors involved in Th1 differentiation (36, 112). Similar effects were observed in Th17 differentiation, MCPIP-1 works cooperatively with roquin to suppress the differentiation of pro-inflammatory Th17 cells (36). IL-17, a cytokine produced by Th17 cells, has been indicated in the pathogenesis of chronic inflammatory and autoimmune diseases such as psoriasis (113). MCPIP-1 is induced by IL-17A *via* the phosphorylation of STAT3 (31) and negatively regulate IL-17-dependent inflammation through the degradation of IL-17A-induced target gene transcripts and IL-17RA mRNA (112). Ablation of MCPIP-1 in keratinocytes resulted in the upregulated expression of transcripts encoding factors related to inflammation and keratinocyte differentiation (114). Similar to the results obtained with Th17 cells, MCPIP-1 was shown to play a role in Th2 cell differentiation by dampening of Gata3 expression through the degradation of Gata3 mRNA (115). Mice lacking MCPIP-1 suffered severe airway inflammation, with increased numbers of airway Th2 cells and elevated level of IL-5 (115). These findings suggest MCPIP-1 has pleiotropic effects that contributes to maintaining homeostasis under inflammatory conditions.

The involvement of MCPIP-1 in adipogenesis was also reported, in which MCPIP-1 was thought to induce p47phox, a critical component of NADPH oxidase that contributes to the increase of ROS, which initiates the sequential differentiation process (116, 117). Other studies, however, reported an opposite effect of MCPIP-1 on adipogenesis (118), which shows that forced expression of MCPIP-1 decreases mRNA levels of the C/EBPβ and PPARγ, two key transcription factors controlling adipogenesis, leading to the impairment of adipogenesis (118). Although the evidence described above suggests that MCPIP-1 may exhibit diverse actions according to normal or pathological conditions and the types of cells, it should be noted that most of the evidence regarding the pleiotropic effects of MCPIP-1 were observed *in vitro*. In addition, most of the studies dissecting the effects of MCPIP-1 were associative and further investigations are warranted to address the pleiotropic effects of MCPIP-1.

## Roles of MCPIP-2, -3, and -4 in the Regulation of Inflammation

Compared to MCPIP-1, the biological roles played by other three members of the Zc3h12 family are less characterized, although it appears that these members are also involved in inflammatory processes. Similar to MCPIP-1, MCPIP-2 has been shown to regulate the course of inflammation. MCPIP-2 participates in the degradation of IL-6 mRNA, resulting in reduced production of IL-6 protein upon stimulation with IL-1β (119). MCPIP-2 also interacts with other known substrates of MCPIP-1 and MCPIP-4, such as the 3’UTR of IER3 mRNA, leading to the degradation of the target mRNAs (119). In a separate study by Huang et al. (120) indicated that IL-6 mRNA is not a direct target of MCPIP-2, which could not exclusively attributable to the different cell line used. In addition, Suzuki et al. (42) showed that MCPIP-2 lacks the miRNA silencing activity, which is attributed to the lack of the proline-rich domain important for this activity. The biological roles of MCPIP-2 remain completely unknown, and further investigations are warranted to address this issue.

MCPIP-3 also contains an RNase domain at the N-terminus before the CCCH-zinc finger domain. Liu et al. (121) showed that MCPIP-3 is able to inhibit the endothelial cell inflammatory response *in vitro* by suppressing NFκB activation in human endothelial cells. Mice with MCPIP-3 deficiency developed hypertrophic lymph nodes and a higher proportion of immature B cells and innate immune cells, particularly macrophages, by regulating IFN signaling (122). Like MCPIP-1, MCPIP-3 is an RNase essential for immune homeostasis, which binds, degrades and regulates mRNAs, such as MCPIP-1 and IL-6, as observed by reduction in luciferase activity (123). Further comparative structural analysis of MCPIP-3 suggests that the RNA substrate is cooperatively recognized by the PIN and Zinc finger domains of MCPIP-3 (123). Unlike MCPIP-1, MCPIP-3 is specifically expressed in macrophages and is transcriptionally controlled by IFN signaling (122). In humans, MCPIP-3 has been linked with chronic immune disorders like psoriasis *via* regulating TNFα and Th1 activation (124, 125). Recently, Liu et al. reported that MCPIP-3 expression is positively associated with psoriasisform lesions, and highly expressed in macrophages and plasmacytoid dendritic cells (126). In the same study, the authors demonstrated that MCPIP-3 may promote TNFα/IL-12 *via* the degradation of MCPIP-1 and IL-6 *via* direct mRNA degradation, contributing to psoriatic skin inflammation. Consistently, mice with MCPIP-3 deficiency are protected from imiquimod-induced psoriasiform lesions. These data suggest that MCPIP-3 could be a potential inhibitory target to treat psoriasis and other autoimmune diseases (126).

MCPIP-4 was originally reported as a putative tumor suppressor that is deregulated in transformed follicular lymphoma in human (127). A single nucleotide polymorphism analysis indicated that MCPIP-4 is associated with the suppression of tumor cell growth both *in vitro* and *in vivo* (128). Similar to MCPIP-1, the expression of MCPIP-4 was markedly induced by TLR ligands through the activation of JNK and NFκB signal pathways, while forced expression of MCPIP-4 inhibited the activation of JNK, ERK, and NFκB signaling in macrophages (129). The latter is achieved by the inhibition of global protein ubiquitination, a key event in the regulation of NFκB activation, suggesting MCPIP-4 is a novel negative feedback regulator of TLR signaling and macrophage activation (129). MCPIP-4 also participates in the degradation of pro-inflammatory mRNAs, such as the mRNAs of IL-2, IL-6, IL-10, TNFα, IER3, and MCPIP-1 (130, 131). Mechanistically, it was demonstrated that MCPIP-4 interacts with MCPIP-1 to form a protein complex, but acts independently in the regulation of IL-6 mRNA degradation (120). To test the *in vivo* effect of MCPIP-4 in determining host immunity, Minagawa et al. generated a model with MCPIP-4 deletion in mice that displayed normal phenotypes under normal condition, but exhibited more activated lymphocytes, particularly Th17 cells, upon inflammatory stimulation (132). In experimental autoimmune encephalitis induced in the MCPIP-4-deficient mice, a higher proportion of Th17 cells with increased IL-17A mRNA levels were observed in the brain of MCPIP-4 deficient mice than did those in MCPIP-4 wild-type mice, suggesting MCPIP-4 may suppress excessive inflammation in the brain by inhibiting the infiltration and activation of Th17 cells in the experimental autoimmune encephalitis (132).

## Conclusions and Perspectives

In this review, we summarized the relevant literature about the role of MCPIP family proteins, in particular MCPIP-1, in the regulation of inflammatory response in different stress conditions. *In vivo* and *in vitro* studies revealed that MCPIP-1 expression by immune and non-immune cells contributes to the resolution of inflammation through distinct cellular and molecular programs. Overall, the expression of MCPIP-1 may be a promising target for the prevention and treatment of inflammatory disorders. Besides its crucial role the regulation of inflammation, MCPIP-1 appeared to play a significant role in diverse cellular functions in a variety of cell types, including macrophages, T cells, MSCs, endothelial progenitor cells and adipocytes, as we discussed above in this review. Macrophages are a major source of active inflammation associated with various chronic inflammatory diseases, such as cardiovascular disease, obesity, atherosclerosis, bone loss, and cancer. Understanding how MCPIP-1 regulates macrophage phenotype and modulates nflammatory response may offer promising opportunities for the development of novel therapeutic approach for these disorders. In addition, MCPIP-1 processes antiviral cellular response by degrading the genomic nucleic acids of both positive-sense and negative-sense RNA viruses and DNA viruses. There is a clear potential for MCPIP-1 to be considered as a therapeutic target to prevent the deleterious effects of cytokine storms caused by SARS−CoV−2 infection, although much remains to be investigated. On the other hand, other MCPIP members appear to be involved in the regulation of inflammatory processes. Additional studies are needed to elucidate the effects of other members on the regulation of inflammation, which would include the crosstalk of the proteins from this family and the mechanisms of their actions, especially those related to the resolution of inflammation. Finally, it should be assessed in the future whether the modulation of these proteins should contribute to the discovery of new pharmacological targets that allow us to design specific strategies to resolve inflammation, especially in the context of acute or chronic inflammatory diseases.

## Author Contributions

ZJ wrote the manuscript. EZ conducted literature collection and summary. JN prepared the final version of the manuscript. CS and PK critically reviewed the manuscript. All authors contributed to the article and approved the submitted version.

## Funding

This work was supported in part by the National Natural Science Foundation of China (Grant No. 81774010).

## Conflict of Interest

The authors declare that the research was conducted in the absence of any commercial or financial relationships that could be construed as a potential conflict of interest.

## Publisher’s Note

All claims expressed in this article are solely those of the authors and do not necessarily represent those of their affiliated organizations, or those of the publisher, the editors and the reviewers. Any product that may be evaluated in this article, or claim that may be made by its manufacturer, is not guaranteed or endorsed by the publisher.
